# The immune regulation and therapeutic potential of the SMAD gene family in breast cancer

**DOI:** 10.1038/s41598-024-57189-6

**Published:** 2024-03-21

**Authors:** Zhuo Chen, Yu Wang, Xiaodi Lu, Hong Chen, Yiran Kong, Liwei Rong, Guonian Wang

**Affiliations:** 1 Department of Anesthesiology, Harbin Medical University Cancer Hospital, Haping Road No. 150, Harbin, 150081 Heilongjiang China; 2 Department of Medical Records, Harbin Medical University Cancer Hospital, Haping Road No. 150, Harbin, 150081 Heilongjiang China; 3 Department of Anesthesiology, The Fourth Affiliated Hospital of Harbin Medical University, Harbin, China; 4 Institute of Cancer Prevention and Treatment, Heilongjiang Academy of Medical Sciences, Harbin, China

**Keywords:** SMAD family genes, Breast cancer, Pathways, Immune, Drug, Cancer, Computational biology and bioinformatics, Diseases

## Abstract

Breast cancer is a serious threat to human health. The transforming growth factor-β signaling pathway is an important pathway involved in the occurrence and development of cancer. The SMAD family genes are responsible for the TGF-β signaling pathway. However, the mechanism by which genes of the SMAD family are involved in breast cancer is still unclear. Therefore, it is necessary to investigate the biological roles of the SMAD family genes in breast cancer. We downloaded the gene expression data, gene mutation data, and clinical pathological data of breast cancer patients from the UCSC Xena database. We used the Wilcox test to estimate the expression of genes of the SMAD family in cancers. And the biological functions of SMAD family genes using the DAVID website. The Pearson correlation method was used to explore the immune cell infiltration and drug response of SMAD family genes. We conducted in biological experiments vitro and vivo. In this study, we integrated the multi-omics data from TCGA breast cancer patients for analysis. The expression of genes of SMAD family was significantly dysregulated in patients with breast cancer. Except for SMAD6, the expression of other SMAD family genes was positively correlated. We also found that genes of the SMAD family were significantly enriched in the TGF-β signaling pathway, Hippo signaling pathway, cell cycle, and cancer-related pathways. In addition, SMAD3, SMAD6, and SMAD7 were lowly expressed in stage II breast cancer, while SMAD4 and SMAD2 were lowly expressed in stage III cancer. Furthermore, the expression of genes of the SMAD family was significantly correlated with immune cell infiltration scores. Constructing a xenograft tumor mouse model, we found that SMAD3 knockdown significantly inhibited tumorigenesis. Finally, we analyzed the association between these genes and the IC50 value of drugs. Interestingly, patients with high expression of SMAD3 exhibited significant resistance to dasatinib and staurosporine, while high sensitivity to tamoxifen and auranofin. In addition, SMAD3 knockdown promoted the apoptosis of BT-549 cells and decreased cell activity, and BAY-1161909 and XK-469 increased drug efficacy. In conclusion, genes of the SMAD family play a crucial role in the development of breast cancer.

## Introduction

Breast cancer is the most common malignant tumor that seriously threatens women’s health^1^. The incidence of breast cancer has increased in the past decade, and breast cancer has become a common tumor threatening women's health. The incidence rate of breast cancer varies from region to region worldwide, but it is on the rise. It is a heterogeneous disease with extensive molecular alterations, which contribute to invasion and metastasis^2–4^. Many studies have revealed that many gene mutations are present in breast cancer cells^5–7^. The classification of breast cancer includes HR+/HER2−, HER2+ (HR+/HR−), and triple-negative breast cancer (TNBC), of which TNBC is the most dangerous. Although the 5-year survival rate is more than 80%, the median overall survival is only 1 year after metastasis^8,9^. Therefore, exploring important genes may be critical to understanding disease progression and finding new therapeutic strategies.

Routine treatment of breast cancer mainly includes three types: endocrine therapy, such as estrogen receptor modulators, and aromatase inhibitors. The second line of treatment consists of cytotoxic chemotherapy drugs, including docetaxel^10^, cyclophosphamide^11^, and doxorubicin^12^. The third line of drugs includes HER2-targeted drugs, such as trastuzumab^13,14^. Only patients with hormone receptor (HR)-positive breast cancer can benefit from endocrine therapy. After introducing tamoxifen in 1977, endocrine therapy entered the treatment guidelines. Tamoxifen is an estrogen receptor modulator that directly acts on estrogen receptors to exert anti-tumor effects.

SMAD is a unique intracellular protein responsible for transforming transforming growth factor-β1 (TGF-β) signaling^15^. The signal transduction induced by the superfamily is transmitted to the nucleus. When TGF-β binds to cell surface serine/threonine kinase receptors, they initiate cellular signaling, which will be then propagated propagate through SMADs^16,17^. Activated SMADs translocate from the cytoplasm to the nucleus, along with transcription factors to activate or inhibit transcription, thereby regulating the expression of target genes. Some studies have shown that SMAD family genes can be divided into three subtypes: receptor-regulated SMADs (R-SMADs, such as SMAD 1/2/3/5/8), common pathway SMADs (co-SMADs, such as SMAD4), and inhibitory SMADs (I-SMADs, such as SMADs 6/7). Each one of them which plays a different role in the pathway^18–20^.

Previous studies have shown the association between SMAD4 deficiency and STING-mediated IFN-I signaling pathway in PDAC and suggested that SMAD4 expression can may be used as a biomarker for predicting response to immunotherapy in PDAC^21^. The incidence of deletion and mutation of tumor suppressor gene SMAD4/DPC4 was 55% in pancreatic cancer. Deactivation or low expression of SMAD4 may affect TGF-β signal transduction and involvement in tumor formation. Moreover, patients with SMAD4 deficiency had a worse prognosis in colorectal cancer. ATAD2 interacts with a new cofactor C/EBP-β combined with TGF-β1/SMAD3 signaling pathway to promote epithelial mesenchymal transition (EMT), ultimately leading to metastasis of esophageal squamous cell carcinoma (ESCC)^22^. In addition, dietary intake of creatine or GATM-mediated synthesis of creatine activated SMAD2 and phosphorylated SMAD3 through monopolar spindle 1 (MPS1), upregulated the expression of Snail and Slug, enhanced cancer cell metastasis, and shortened the survival of mice^23^. Other studies indicated that increased EZH2 expression abnormally increased SMAD3 methylation, thereby activating SMAD3. Clinically, researchers demonstrated that SMAD3 methylation was significantly associated with poor survival of patients with breast cancer^24^. In terms of treatment, targeted methylation of SMAD3 can inhibit tumor cell metastasis.

In breast cancer, dysfunction of the SMAD gene family is closely related to tumorigenesis and response to treatment^25–28^. Abnormal expression of the *SMAD* gene family may dysregulate signaling pathways, thereby leading to uncontrolled proliferation, and inhibiting the apoptosis of breast cancer cells. SMAD regulates TGF-β-mediated EMT, and its abnormal activation may play a role in the invasion and metastasis of breast cancer. Breast cancer stem cells play a key role in tumorigenesis, recurrence, and treatment resistance. The SMAD signaling pathway regulates the behavior of breast cancer stem cells and affects the stemness properties and treatment response of breast cancer. Studies have shown that drugs targeting the TGF-β/SMAD signaling pathway may have therapeutic potential for some patients with breast cancer. The expression levels of the SMAD family of genes in breast cancer tissues can be used as potential biomarkers to predict patients’ prognosis and treatment response.

Therefore, using data from TCGA breast cancer patients’ multi-omics, we investigated the roles of SMAD in breast cancer from multiple perspectives. We not only analyzed the association between the expression of SMAD and that of other genes but also analyzed its association with immune cell infiltration and IC50 values of drugs. We experimentally validated the role of SMAD3 in drug resistance and immune cell infiltration. Our results showed that genes of the SMAD family play an important role in the development of breast cancer.

## Data and methods

### The gene expression profile of breast cancer

We downloaded the gene expression data, gene mutation data, and clinical pathological data of patients with breast cancer from the UCSC Xena database (https://xena.ucsc.edu/). Gene expression data included 1097 breast cancer samples and 114 normal samples. Among them, 202 patients were in stage I, 690 patients were in stage II, 276 patients were in stage III, and 22 patients were in stage IV. The gene mutation data included 791 breast cancer samples and 40,543 gene mutations. We used the log-rank test to analyze the prognostic efficacy of genes of the SMAD family according to the GEPIA2 data resources^29^.

### Analysis of immune cell infiltration scores

We downloaded the immune score, ESTIMATE score, and stromal score of patients with breast cancer from the ESTIMATE database (https://bioinformatics.mdanderson.org/estimate/). Moreover, we downloaded immune cell gene sets and calculated the immune cell infiltration score of patients with breast cancer based on the single sample gene set enrichment analysis (ssGSEA) method^30^. Furthermore, the Pearson correlation method was used to analyze the associations between the expression of SMAD family genes and immune cell infiltration scores.

### Xenograft mouse model

Animal experiments were conducted following the Guidelines for the Care and Use of Laboratory Animals published by the National Institutes of Health and were approved by the Ethics Committee of Harbin Medical University. BALB/c mice (female, 4–6 weeks, 16–18 g) were housed in a pathogen-free facility. 4T1 cells with smad3 deletion (5 × 10^6^) and control cells were subcutaneously injected into the right flanks of mice. The length (L) and width (W) of tumors were measured every 3 days. Then, the volume of each tumor was calculated using the formula V = 1/2L*W2. On day 15, mice were euthanized using the cervical dislocation method under anesthesia (0.7% sodium pentobarbital), and tumors were harvested and weighed. Tumor volume (mm^3^) in each group was graphed over time to monitor tumor growth and assess the synergistic effect of SMAD deletion in vivo.

### Immunochemistry analysis

Mouse xenograft tumor tissues were excised and fixed in 10% formalin and paraffin-embedded sections were prepared. For immunochemistry analysis, sections were deparaffinized, hydrated, and boiled for 10 min in 10 mM citrate buffer (pH 6.0), followed by cooling for 20 min at RT. Sequential blocking with 3% BSA was conducted for 1 h to prevent unspecific antibody binding. Staining was performed using corresponding antibodies at 4 °C overnight. Subsequently, a biotinylated secondary antibody was employed. Streptavidin-HRP conjugates and DAB Kit were used as the chromogenic substrate. The sections were then counterstained with haematoxylin.

### Drug response analysis

We downloaded and obtained IC50 values for 58 cell lines and 860 drugs from GDSC database (http://www.cancerrxgene.org/)^31,32^ and analyzed the correlation between SMAD family gene expression and drug IC50 value using the Pearson correlation method.

### CCK8 cell viability assay

BT-549 cells were cultured in RPMI-1640 medium containing 10% fetal bovine serum, 10 μg/mL insulin, and 1% penicillin/streptomycin. Cells were incubated in a constant-temperature humidified incubator at 37 °C and 5% CO_2_. For subculture, cells were digested using 0.25% trypsin. BT-549 cells were seeded in 96-well plates with a cell density of 5 × 10^3^ cells per well. After incubating overnight, cells were attached and grown in wells. Then, the RPMI 1640 medium was removed and replaced by fresh RPMI-1640 medium containing different concentrations of BAY-1161909 or XK-469. After 24 h of treatment, 10 μL of CCK-8 reagent was added to each well in the dark. Then, the 96-well plate was wrapped with foil and put in the incubator. After 2 h, the absorbance OD values were measured at 450 nm using a microplate reader, and the cell survival rate was calculated according to the following formula.

$$ {\text{Cell survival rate}}\% = \left( {{\text{absorbance OD value of experimental group}} - {\text{absorbance of the blank group}}} \right) \div \left( {{\text{absorbance OD value of the control group}} - {\text{absorbance of the blank group}}} \right) \times {1}00\% $$

### Apoptosis analysis by flow cytometry

Cells were seeded in six-well plates at a density of 2 × 10^5^ cells/well (in triplicate) and grown overnight. Then, cells were treated with different concentrations of BAY-1161909 or XK-469. After 24 h, cells were harvested and then incubated with PI and Annexin V-FITC using an apoptosis detection kit following the manufacturers’ instructions. Finally, cells were measured using flow cytometry (Becton Dickinson, FACS Verse, USA). FlowJo7.6 software was used for data analysis.

### Ethics statement

We confirmed that all methods are reported in accordance with ARRIVE guidelines (https://arriveguidelines.org). Animal experiments were conducted following the Guidelines for the Care and Use of Laboratory Animals published by the National Institutes of Health and were approved by the Ethics Committee of Harbin Medical University and animals were euthanized following the 2020 AVMA Guidelines. The data used in the article is from the TCGA database and is publicly available.

## Results

### The multi-omics analysis of genes of the SMAD family

**Figure 1:**
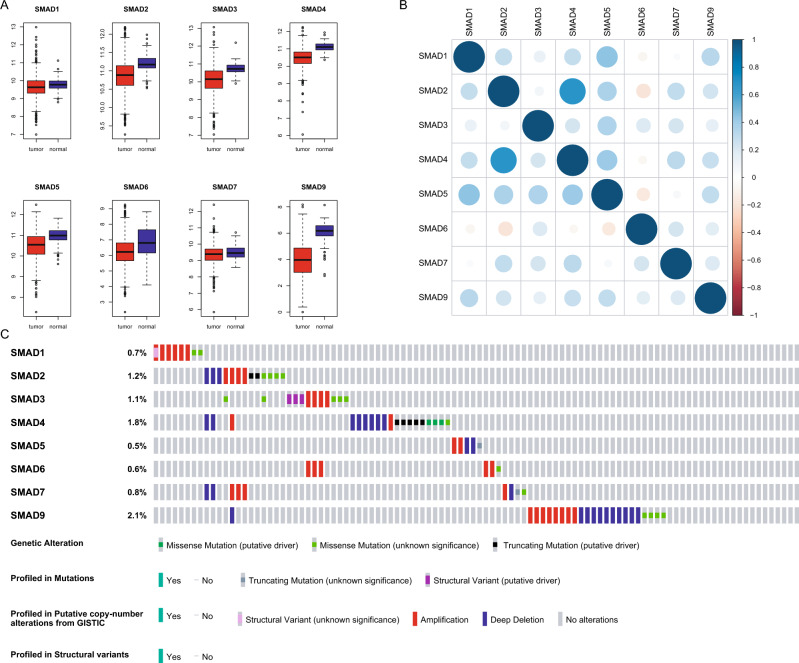
The multi-omics analysis of genes of the SMAD family. (**A**) The differential expression of SMAD family genes in breast cancer samples and normal samples. (**B**) The expression correlation of these genes. (**C**) The genomic variations of SMAD family genes.

We analyzed the expression of genes of the SMAD family in patients with breast cancer from the TCGA database. These genes were significantly dysregulated in cancer tissue (Fig. 1A, Table S1). There was a greater difference in the expression of SMAD2, SMAD3, SMAD4, SMAD5, and SMAD9 between cancer and normal samples. Then, we performed correlation analysis on the expression of genes. Except for the SMAD6 gene, other genes of the SMAD family were positively correlated in patients with breast cancer. In addition, the expression of SMAD3 was positively correlated with that of SMAD6, while SMAD6 expression was negatively correlated with the expression of other genes (Fig. 1B). Log-rank test showed that the expression of genes of the SMAD family could not significantly predict the overall survival (OS) and disease-free survival (DFS) of patients (Figs. S1, S2).

Furthermore, we analyzed the genetic changes in SMAD family genes in patients with breast cancer from the cBioportal database (https://www.cbioportal.org/). Genetic changes in the SMAD9 gene were observed in 2.1% of patients with breast cancer. SMAD4, SMAD2, SMAD3, SMAD7, SMAD1, SMAD6, and SMAD5 showed genetic variation in 1.8%, 1.2%, 1.1%, 0.8%, 0.7%, 0.6%, and 0.5% of patients with breast cancer, respectively (Fig. 1C).

### The functional analysis of SMAD family genes

**Figure 2:**
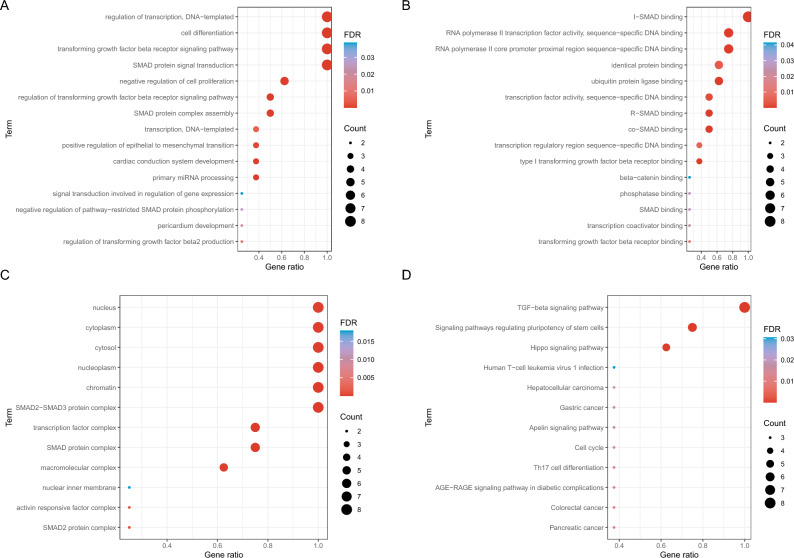
The functional enrichment analysis of genes of the SMAD family: (**A**) biological process of gene ontology, (**B**) molecular function of gene ontology, (**C**) cellular component of gene ontology, and (**D**) Kyoto Encylopaedia of Genes and Genomes pathways.

Next, we explored the functions of SMAD family genes (Fig. 2). The SMAD family genes were significantly enriched in cell differentiation, negative regulation of cell proliferation, and regulation of TGF-β receiver signaling pathway. In the pathway enrichment analysis, the SMAD family genes were significantly enriched in the TGF-β signaling pathway, Hippo signaling pathway, cell cycle, and cancer-related pathways, such as gastric cancer and pancreatic cancer. In the TGF-β signaling pathway, TGFβRI, and TGFβRII, ACVR2A, and ACVR2B promoted the expression of SMAD family genes (including SMAD2, SMAD3, and SMAD4), thereby affecting cell cycle and including stem promoter phenotype (Fig. S3).

### The expression of SMAD family genes in different stages of breast cancer

**Figure 3:**
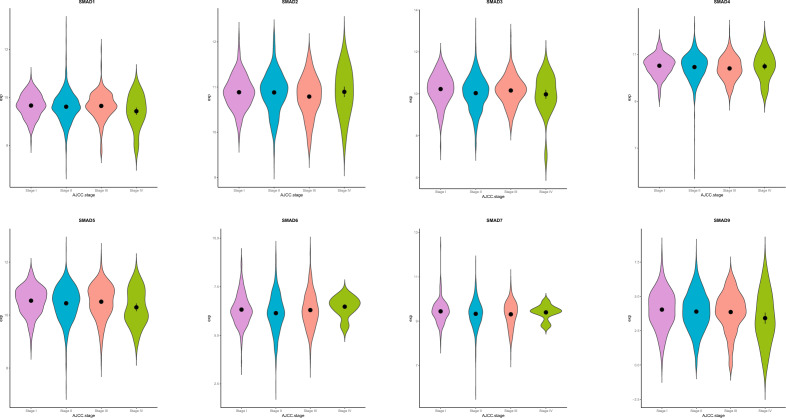
The expression of SMAD family genes among different stages of breast cancer patients.

We also analyzed the expression of SMAD family genes in different TNM stages of breast cancer (Fig. 3). Except for SMAD1 and SMAD9 genes, other SMAD family genes were significantly and differentially expressed at a certain stage. SMAD3, SMAD6, and SMAD7 were significantly downregulated in stage II (p-value = 0.002 for SMAD3, p-value = 0.041 for SMAD6, and p-value = 0.045 for SMAD7). SMAD4 was significantly downregulated in stage III (p-value = 0.011), and SMAD2 was non-significantly downregulated in stage III (p-value = 0.070). Similarly, we found that the SMAD5 tended to be downregulated in stage IV (p-value = 0.085). These results showed that the SMAD family genes were differentially expressed in different stages of breast cancer.

### The correlation between immune cell infiltration and expression of SMAD family genes

**Figure 4:**
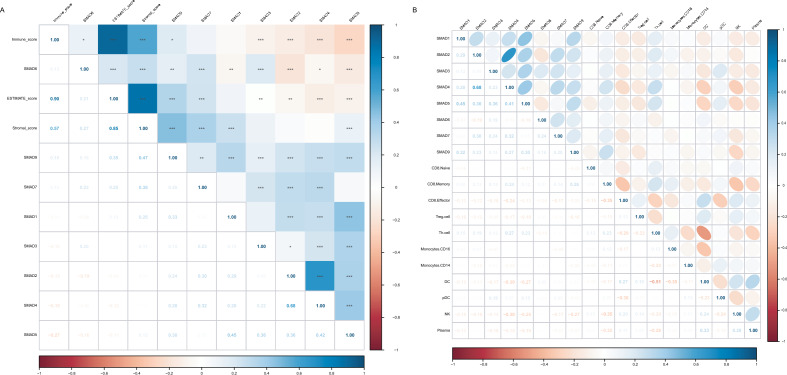
The correlation between the expression of genes of the SMAD family and immune cell infiltration score. (**A**) The scores of the immune score, stromal score, and estimate score. (**B**) The immune cell infiltration score in breast cancer samples.

Human immune system is deeply involved in the development of breast cancer. Firstly, we used the ESTIMATE algorithm to assess the association of SMAD family genes with immune score, ESTIMATE score, and stromal score (Fig. 4A). We found that the expression of SMAD9 and SMAD6 genes was significantly and positively correlated with immune score, whereas the expression of SMAD2, SMAD3, SMAD4, and SMAD5 was significantly and negatively correlated with immune score (p-value < 0.05). Similarly, the expression of SMAD9 and SMAD7 was significantly and positively correlated with the ESTIMATE score, whereas the expression of SMAD2, SMAD3, SMAD4, and SMAD5 was significantly and negatively correlated with this score (p-value < 0.05). Meanwhile, SMAD1, SMAD6, SMAD7, and SMAD9 were significantly positively correlated with the stromal score (p-value < 0.05). These results indicated that the expression of SMAD2, SMAD3, SMAD4, and SMAD5 was more closely correlated with the immune response.

Furthermore, we explored the association between the expression of SMAD family genes and immune cell infiltration scores (Fig. 4B). The expression of SMAS2, SMAD3, SMAD4, and SMAD5 were significantly and positively correlated with the cell infiltration scores of CD8 memory cells and helper T cells, while significantly negatively and correlated with the infiltration scores of CD8 effector cells, regulatory T cells, dendritic cells, natural killer cells, and plasma cells. The expression of SMAS6, SMAD7, and SMAD9 showed the opposite to pattern, and they were significantly and positively correlated with the infiltration of dendritic cells. These results suggest that the SMAD family of genes regulates the immune regulation process of cancer.

### The functions of SMAD3 in tumorigenesis

**Figure 5:**
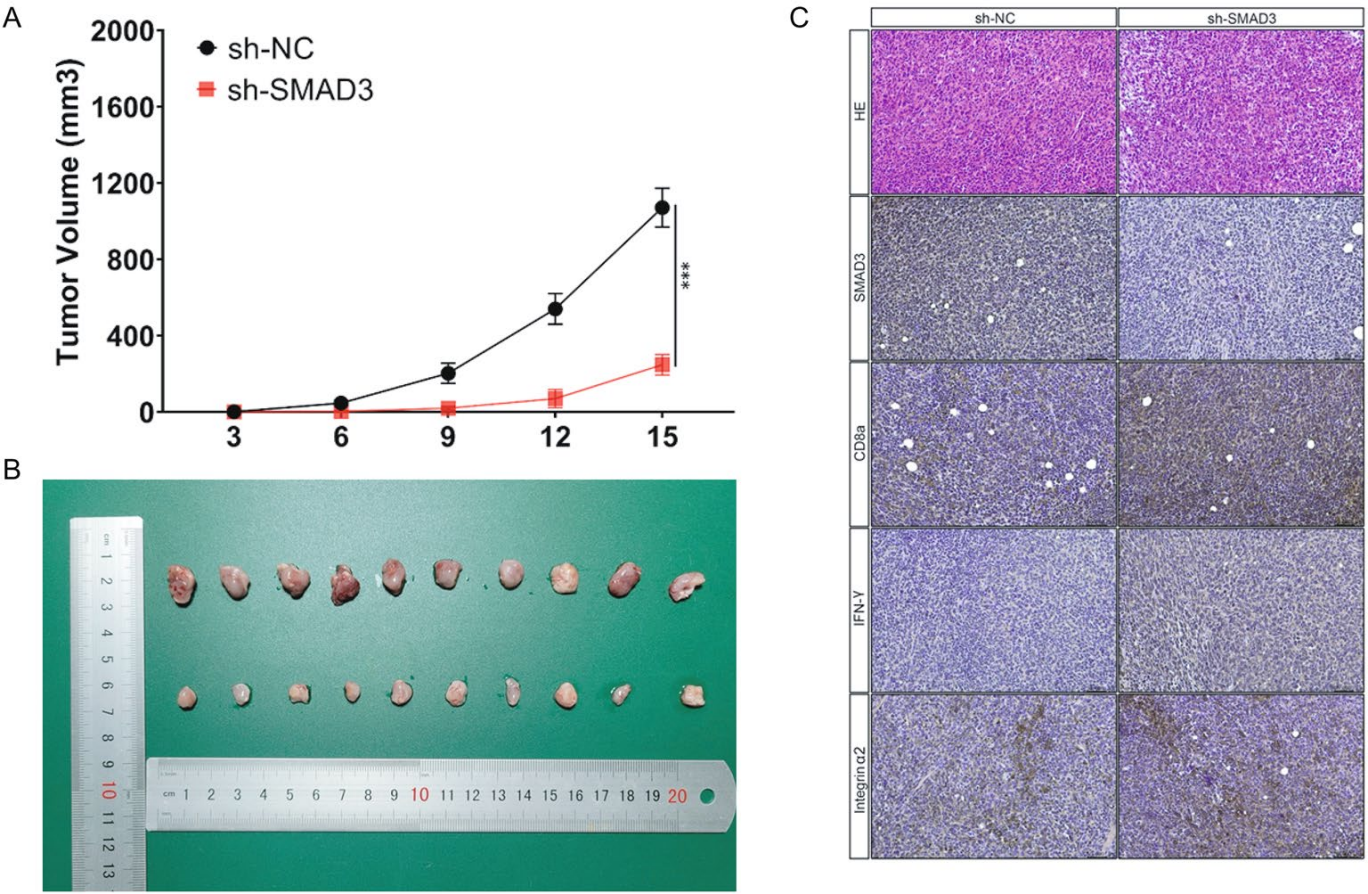
The correlation between SMAD3 and immune cell infiltration score. (**A**) SMAD3 knock-down suppressed tumor growth of breast cancer. (**B**,**C**) Representative micrographs of Immunofluorescence staining for SMAD3, CD8a, IFN-γ and Integrin α2 foci.

We found that the expression of SMAD3 was correlated with the immune response, including CD8 memory cells and helper T cells. We investigated the effect of SMAD3 knockdown in vivo. SMAD3 knockdown 4T1 cells were subcutaneously injected into mice to construct a xenograft tumor model in mice. SMAD3 knockdown significantly inhibited tumorigenesis (Fig. 5). SMAD3 knockdown suppressed breast cancer growth. The results suggested the synergistic inhibitory effect of SMAD3 knockdown on tumor growth in vivo. Together, these findings demonstrated that SMAD3 knockdown inhibited tumor immune escape in vivo.

### The drug sensitivity correlation analysis of SMAD family genes

**Figure 6:**
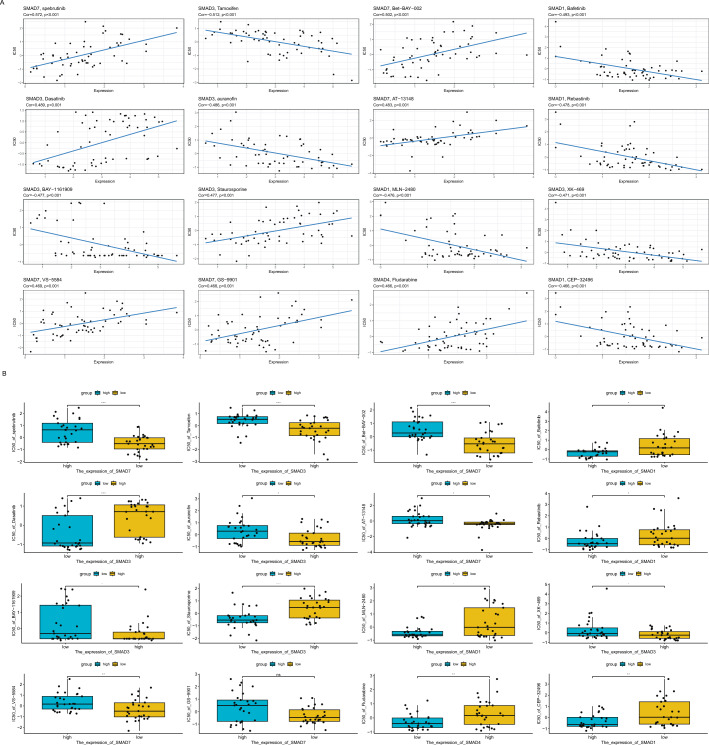
The drug sensitivity correlation analysis of SMAD family genes. (**A**) The scatter plot. (**B**) The box plot.

Finally, we assessed the association between the expression of SMAD family genes and drug sensitivity and heterogeneity (Fig. 6). We downloaded the IC50 values of 860 drugs from the GDSC database. Patients with high expression of SMAD1 showed significantly high sensitivity to bafetinib, rebastinib, MLN-2480, and CEP-32496. Patients with high expression of the SMAD3 exhibited significant resistance to dasatinib and staurosporine, but high sensitivity to tamoxifen, auranofin, BAY-1161909, and XK-469. Patients with high expression of SMAD7 gene exhibited significant resistance to spebrutinib, Bet-BAY-002, AT-13148, VS-5584, and GS-9901. These results indicated that SMAD family genes were associated with anti-cancer drugs.

### The effects of SMAD3 on the apoptosis of breast cancer cells

Based on the above results, we found that the expression of SMAD3 was significantly associated with the immune cell infiltration score and the IC50 value of breast cancer drugs. To investigate whether SMAD3 knockdown can synergistically affect the tolerance of breast cancer cells to BAY-1161909 or XK-469, we first investigated the inhibitory effects of individual drugs.

**Figure 7:**
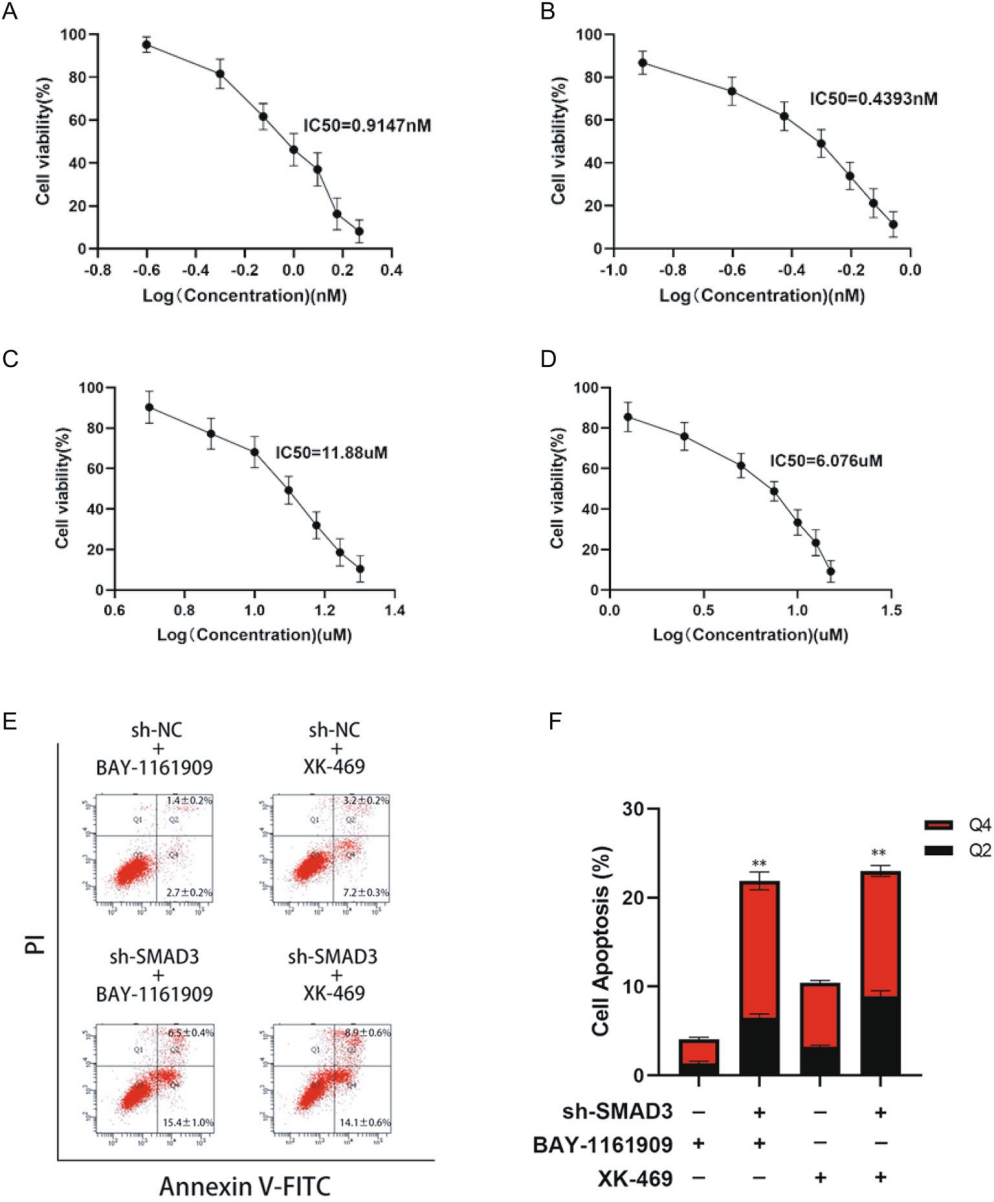
SMAD3 knock-down affects the sensitivity of breast cancer cells to BAY-1161909 and XK-469. (**A**) BT-549 cells were sensitive to BAY-1161909 treatment (IC50 = 0.9147 Nm). (**B**) BT-549 cells were sensitive to XK-469 treatment (IC50 = 11.88 μM). (**C**,**D**) Knock-down SMAD3 could enhance the sensitivity of BT-549 to both BAY-1161909 (IC50 = 0.4393 nM) and XK-469 (IC50 = 6.067 μM). (**E**,**F**) Annexin V/PI staining and flow cytometry analysis of BT-549 cells with different dose of BAY-1161909 or XK-469 treatment for 24 h.

The condition of BT-459 cells after SMAD3 knockdown was analyzed by CCK8 and flow cytometry. The results showed that SMAD3 knockdown promoted the apoptosis of BT-549 cells and decreased cell activity, whereas BAY-1161909 and XK-469 increased drug efficacy (Fig. 7A–D). Annexin V/PI staining and flow cytometry analysis were conducted for BT-549 cells 24 h after receiving different doses of BAY-1161909 or XK-469. We also performed Annexin V-fluorescein isothiocyanate (FITC)/propidium iodide (PI) staining, which is frequently used to measure apoptosis. SMAD3 knockdown enhanced BAY-1161909- and XK-469-mediated apoptosis (Q2 late apoptosis and Q4 early apoptosis) (Fig. 7E,F).

### The genomic variation analysis associated with SMAD3

**Figure 8:**
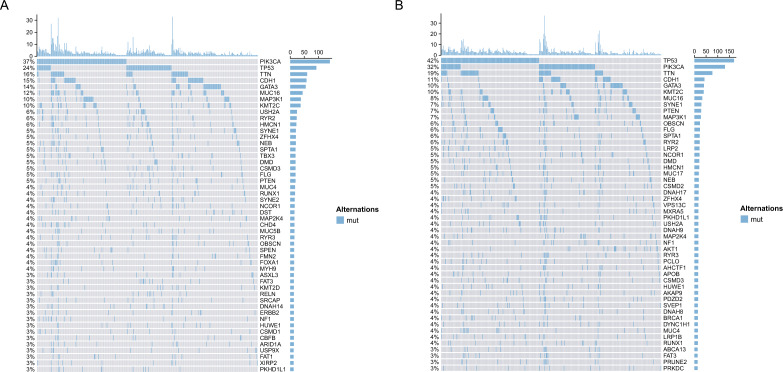
The genomic variation analysis according to the expression of SMAD3. (**A**) Breast cancer patients with high expression of it. (**B**) Breast cancer patients with low expression of it.

Based on the above research, we found that the expression of SMAD3 was not only associated with the immune cell infiltration score but was also significantly associated with the IC50 value of breast cancer drugs. Based on SMAD3 gene expression, patients with breast cancer were divided into the high-expression group and the low-expression group (Fig. 8). To analyze the effect of SMAD3 gene expression on patients’ genomes, we explored the gene mutation distribution profiles of the two groups. Mutations in the TP53, PIK3CA, TTN, CDH1, GATA3, KMT2C, MUC16, SYNE1, PTEN, and MAP3K1 genes were present in at least 7% of patients with low expression of SMAD3, while mutations in the PIK3CA, TP53, TTN, CDH1, GATA3, MUC16, MAP3K1, and KMT2C genes were present in at least 10% of patients with high expression of SMAD3. In summary, there were differences in frequent gene mutations between the high- and low-expression groups, but most gene mutations were similar. In addition, we analyzed the relationship between SMAD3 gene expression and tumor mutation load. SMAD3 gene expression was not related to tumor mutation load in patients with breast cancer, which also verifies the results of gene mutation distribution map analysis.

## Discussion

In this study, we used the multi-omics data of patients with breast cancer from TCGA for analysis. Except for SMAD6, the expression of other SMAD family genes was positively correlated. We also found that the SMAD family genes were significantly enriched in cancer-related pathways. In addition, the expression of SMAD family genes was significantly correlated with immune cell infiltration scores. We demonstrated that SMAD3 knockdown inhibited tumor immune escape in vivo. We also analyzed the association between the SMAD family genes and the IC50 value of drugs. Patients with high expression of SMAD3 showed significant resistance to dasatinib and staurosporine, while high sensitivity to tamoxifen and auranofin. Furthermore, we analyzed the association between the expression of SMAD3 and anesthetic drugs. In addition, we confirmed that SMAD3 knockdown had synergistic effects on BAY-1161909- and XK-469-induced apoptosis of breast cancer cells. Our results indicated that SMAD family genes play an important role in breast cancer.

We found a significant negative correlation between SMAD3 expression and immune score or ESTIMATE score. Moreover, SMAD3 expression was significantly and positively correlated with the infiltration scores of CD8 memory cells and helper T cells while negatively correlated with the infiltration scores of CD8 effector cells, regulatory T cells, dendritic cells, natural killer cells, and plasma cells. Chung et al*.* found that SMAD3 activation in neutrophil TANs was associated with increased N2 phenotype and poor prognosis of NSCLC, while SMAD3 inhibition promoted the polarization of TANs to the anti-tumor N1 phenotype, thereby inhibiting the development of lung cancer^33^. TGF-β enhanced PD-1 expression in an SMAD3-dependent manner. Therefore, TGF-β regulates the expression of several genes to reduce CTL differentiation and function in cytotoxic T cells^34,35^. However, in our study, SMAD3 knockdown suppressed breast cancer growth, which demonstrated that SMAD3 knockdown can inhibit tumor immune escape in vivo.

At the same time, we found that SMAD4 similarly affects the immune. The TGF-β induced SMAD3/SMAD4 complex-mediated activation of activated PKA to trigger carboxyl terminal SRC kinase (CSK)-mediated inhibition of proximal TCR signal and prevent unexpected T cell activation. The expression of TGFBR1 in NK cells can enhance cancer cell metastasis and accelerate the growth of fibrosarcoma, indicating that TGF-β activates SMAD4 to inhibit NK cell-mediated monitoring in cancer metastasis^36^.

Endocrine therapy for breast cancer has a history of more than 100 years and plays an indispensable role in the treatment of HR-positive patients. Tamoxifen is the most widely used drug for endocrine therapy, which mainly competitively binds to tumor cell ER, thereby preventing the promoting effect of estrogen on tumor cell growth and proliferation^37–39^. We found that patients with high expression of SMAD3 showed significantly high sensitivity to tamoxifen and auranofin. Prahlad et al. found that auranofin, an FDA-approved thioredoxin reductase inhibitor, caused specific cell death and impaired the growth of TNBC cells^40^. Joo-Eun et al. found that mesupron combined with auranofin may exhibit synergistic anti-cancer effects^41^. There are also studies indicating that combined treatment with auranofin and trametinib synergistically induces apoptosis in breast cancer cells^42^. In addition, we also found that patients with high expression of SMAD3 showed significant resistance to dasatinib and staurosporine. Single-agent dasatinib has limited activity in patients with triple-negative breast cancer^43^. We also found that SMAD3 knockdown promoted the apoptosis of BT-549 cells and decreased cell activity, whereas BAY-1161909 and XK-469 increased drug efficacy. Furthermore, SMAD3 knockdown enhanced BAY-1161909- and XK-469-induced apoptosis. The synergy between dasatinib and doxorubicin warranted the re-assessment of dasatinib as an effective agent in multi-drug regimens for treating invasive breast cancers^44^.

## Conclusion

We used multi-omics data of breast cancer patients from TCGA to explore the functions of SMAD family genes. We found that the expression of other SMAD family genes was positively correlated. The SMAD family genes were significantly enriched in cancer-related pathways. In addition, the expression of SMAD family genes was significantly correlated with immune cell infiltration scores. Furthermore, the high expression of SMAD3 was associated with significant resistance to dasatinib and staurosporine while high sensitivity to tamoxifen and auranofin. In addition, we explored the genomic characteristics of SMAD3. Our results indicated that SMAD family genes play an important role in breast cancer.

### Supplementary Information

Supplementary Figures.

Supplementary Table S1.

## Data Availability

The datasets used and/or analyzed during the current study available from the corresponding author on reasonable request.

## References

[1] Delman KA (2020). Introducing the “Virtual Tumor Board” series in CA: A Cancer Journal for Clinicians. CA Cancer J. Clin..

[2] Cancer Genome Atlas Network (2012). Comprehensive molecular portraits of human breast tumours. Nature.

[3] Badve S, Gokmen-Polar Y (2015). Tumor heterogeneity in breast cancer. Adv. Anat. Pathol..

[4] Januskeviciene I, Petrikaite V (2019). Heterogeneity of breast cancer: The importance of interaction between different tumor cell populations. Life Sci..

[5] Stemke-Hale K (2008). An integrative genomic and proteomic analysis of PIK3CA, PTEN, and AKT mutations in breast cancer. Cancer Res..

[6] Kuchenbaecker KB (2017). Risks of breast, ovarian, and contralateral breast cancer for BRCA1 and BRCA2 mutation carriers. JAMA.

[7] Mavaddat N (2010). Genetic susceptibility to breast cancer. Mol. Oncol..

[8] Derakhshan F, Reis-Filho JS (2022). Pathogenesis of triple-negative breast cancer. Annu. Rev. Pathol..

[9] Verhoog LC (1998). Survival and tumour characteristics of breast-cancer patients with germline mutations of BRCA1. Lancet.

[10] Lyseng-Williamson KA, Fenton C (2005). Docetaxel: A review of its use in metastatic breast cancer. Drugs.

[11] Jones SE (2006). Phase III trial comparing doxorubicin plus cyclophosphamide with docetaxel plus cyclophosphamide as adjuvant therapy for operable breast cancer. J. Clin. Oncol..

[12] Lankelma J (1999). Doxorubicin gradients in human breast cancer. Clin. Cancer Res..

[13] Maximiano S (2016). Trastuzumab in the treatment of breast cancer. BioDrugs.

[14] Tokunaga E (2006). Trastuzumab and breast cancer: Developments and current status. Int. J. Clin. Oncol..

[15] Derynck R, Zhang YE (2003). Smad-dependent and Smad-independent pathways in TGF-beta family signalling. Nature.

[16] Hu HH (2018). New insights into TGF-beta/Smad signaling in tissue fibrosis. Chem. Biol. Interact..

[17] Moustakas A, Souchelnytskyi S, Heldin CH (2001). Smad regulation in TGF-beta signal transduction. J. Cell Sci..

[18] Kretzschmar M (2000). Transforming growth factor-beta and breast cancer: Transforming growth factor-beta/SMAD signaling defects and cancer. Breast Cancer Res..

[19] Kang Y (2005). Breast cancer bone metastasis mediated by the Smad tumor suppressor pathway. Proc. Natl. Acad. Sci. USA.

[20] Papageorgis P (2010). Smad signaling is required to maintain epigenetic silencing during breast cancer progression. Cancer Res..

[21] Xiong W (2022). Smad4 deficiency promotes pancreatic cancer immunogenicity by activating the cancer-autonomous DNA-sensing signaling axis. Adv. Sci. (Weinh.).

[22] Cao LJ (2021). ATAD2 interacts with C/EBPbeta to promote esophageal squamous cell carcinoma metastasis via TGF-beta1/Smad3 signaling. J. Exp. Clin. Cancer Res..

[23] Zhang L (2021). Creatine promotes cancer metastasis through activation of Smad2/3. Cell Metab..

[24] Huang C (2022). EZH2-triggered methylation of SMAD3 promotes its activation and tumor metastasis. J. Clin. Investig..

[25] Massague J (2012). TGFbeta signalling in context. Nat. Rev. Mol. Cell Biol..

[26] Moustakas A, de Herreros AG (2017). Epithelial–mesenchymal transition in cancer. Mol. Oncol..

[27] Zhang Y, Alexander PB, Wang XF (2017). TGF-beta family signaling in the control of cell proliferation and survival. Cold Spring Harb. Perspect. Biol..

[28] Zhang Y (2017). NOTCH1 signaling regulates self-renewal and platinum chemoresistance of cancer stem-like cells in human non-small cell lung cancer. Cancer Res..

[29] Tang Z (2019). GEPIA2: An enhanced web server for large-scale expression profiling and interactive analysis. Nucleic Acids Res..

[30] Barbie DA (2009). Systematic RNA interference reveals that oncogenic KRAS-driven cancers require TBK1. Nature.

[31] Iorio F (2016). A landscape of pharmacogenomic interactions in cancer. Cell.

[32] Yang W (2013). Genomics of Drug Sensitivity in Cancer (GDSC): A resource for therapeutic biomarker discovery in cancer cells. Nucleic Acids Res..

[33] Chung JY (2023). Smad3 is essential for polarization of tumor-associated neutrophils in non-small cell lung carcinoma. Nat. Commun..

[34] Nixon BG (2023). TGFbeta control of immune responses in cancer: A holistic immuno-oncology perspective. Nat. Rev. Immunol..

[35] Wu F (2021). Signaling pathways in cancer-associated fibroblasts and targeted therapy for cancer. Signal Transduct. Target. Ther..

[36] Xue VW (2020). Transforming growth factor-beta: A multifunctional regulator of cancer immunity. Cancers (Basel).

[37] Jaiyesimi IA (1995). Use of tamoxifen for breast cancer: Twenty-eight years later. J. Clin. Oncol..

[38] Osborne CK (1998). Tamoxifen in the treatment of breast cancer. N. Engl. J. Med..

[39] Wiebe VJ (1993). Tamoxifen resistance in breast cancer. Crit. Rev. Oncol. Hematol..

[40] Raninga PV (2020). Therapeutic cooperation between auranofin, a thioredoxin reductase inhibitor and anti-PD-L1 antibody for treatment of triple-negative breast cancer. Int. J. Cancer.

[41] Lee JE (2017). Synergistic induction of apoptosis by combination treatment with mesupron and auranofin in human breast cancer cells. Arch. Pharm. Res..

[42] Joo MK (2021). Combined treatment with auranofin and trametinib induces synergistic apoptosis in breast cancer cells. J. Toxicol. Environ. Health A.

[43] Finn RS (2011). Dasatinib as a single agent in triple-negative breast cancer: Results of an open-label phase 2 study. Clin. Cancer Res..

[44] Pichot CS (2009). Dasatinib synergizes with doxorubicin to block growth, migration, and invasion of breast cancer cells. Br. J. Cancer.

