# Supplementary material for: The immune regulation and therapeutic potential of the SMAD gene family in breast cancer
Source: Sci Rep. 2024 Mar 21;14:6769. doi: 10.1038/s41598-024-57189-6 (PMC10958012; doi:10.1038/s41598-024-57189-6)
Supplement: Supplementary file 1 — Supplementary Figures. [file 41598_2024_57189_MOESM1_ESM.docx]

**Supplementary materials**

**The immune regulation and therapeutic potential of the SMAD gene family in breast cancer**

Zhuo Chen^1^, Yu Wang^1^, Xiaodi Lu^1^, Hong Chen^1^, Yiran Kong^1^, Liwei Rong^2^, Guonian Wang^3,4,^ *

^1^ Department of Anesthesiology, Harbin Medical University Cancer Hospital, Haping Road No. 150, Harbin 150081, Heilongjiang, China

^2^ Department of medical records, Harbin Medical University Cancer Hospital, Haping Road No. 150, Harbin 150081, Heilongjiang, China

^3^ Department of Anesthesiology, The Fourth Affiliated Hospital of Harbin Medical University, Harbin, China

^4^ Institute of Cancer Prevention and Treatment, Heilongjiang Academy of Medical Sciences, Harbin, China

^*^ **Corresponding authors:**
Guonian Wang, PhD, Department of Anesthesiology, The Fourth Affiliated Hospital of Harbin Medical University, Harbin, China. Institute of Cancer Prevention and Treatment, Heilongjiang Academy of Medical Sciences, Harbin, China.

Email: wangguonian609cn@aliyun.com


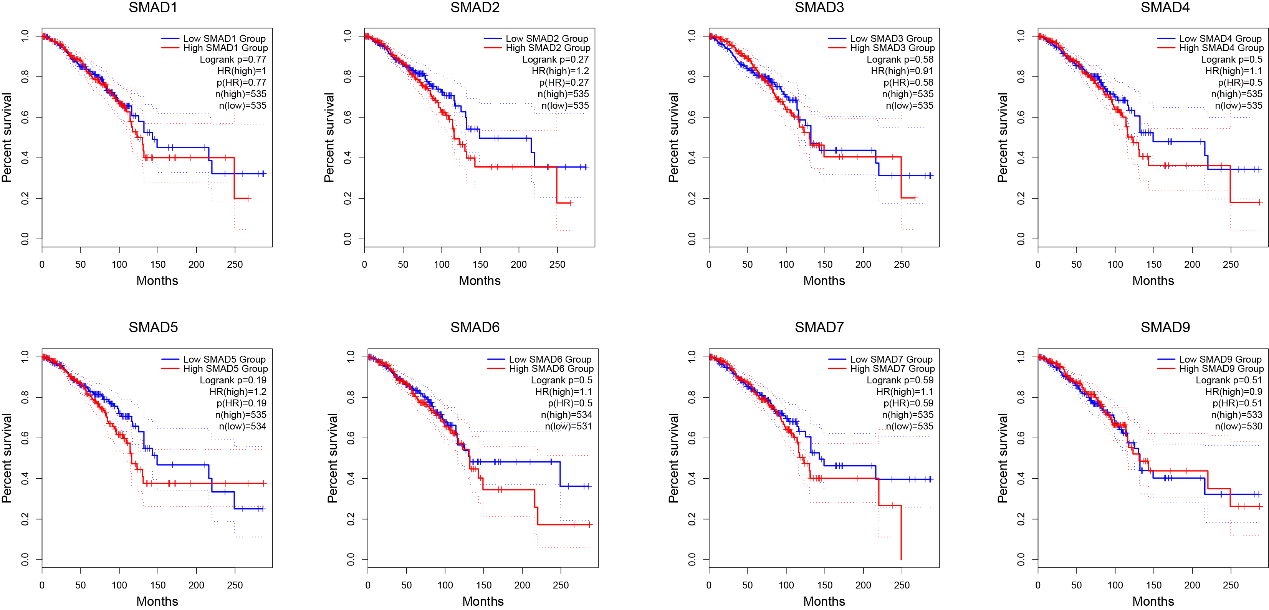


**Figure S1.** **Overall survival analysis for breast cancer patients according to the expression of SMAD family genes.**


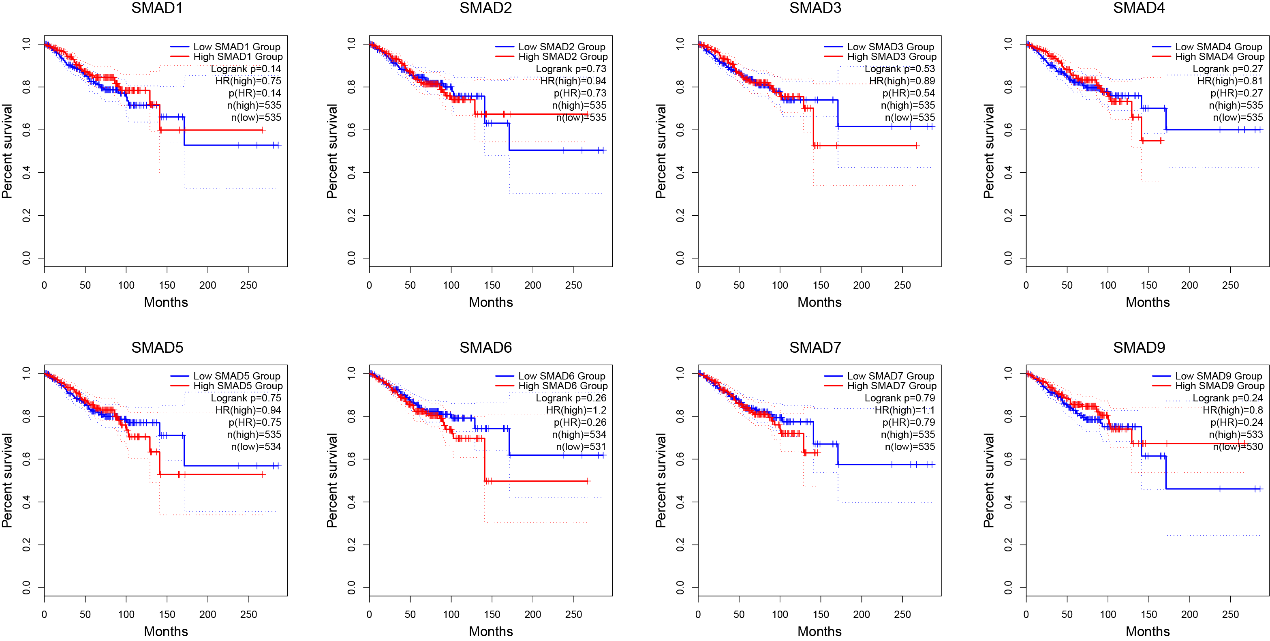


**Figure S2.** **Disease-free survival analysis for breast cancer patients according to the expression of SMAD family genes.**


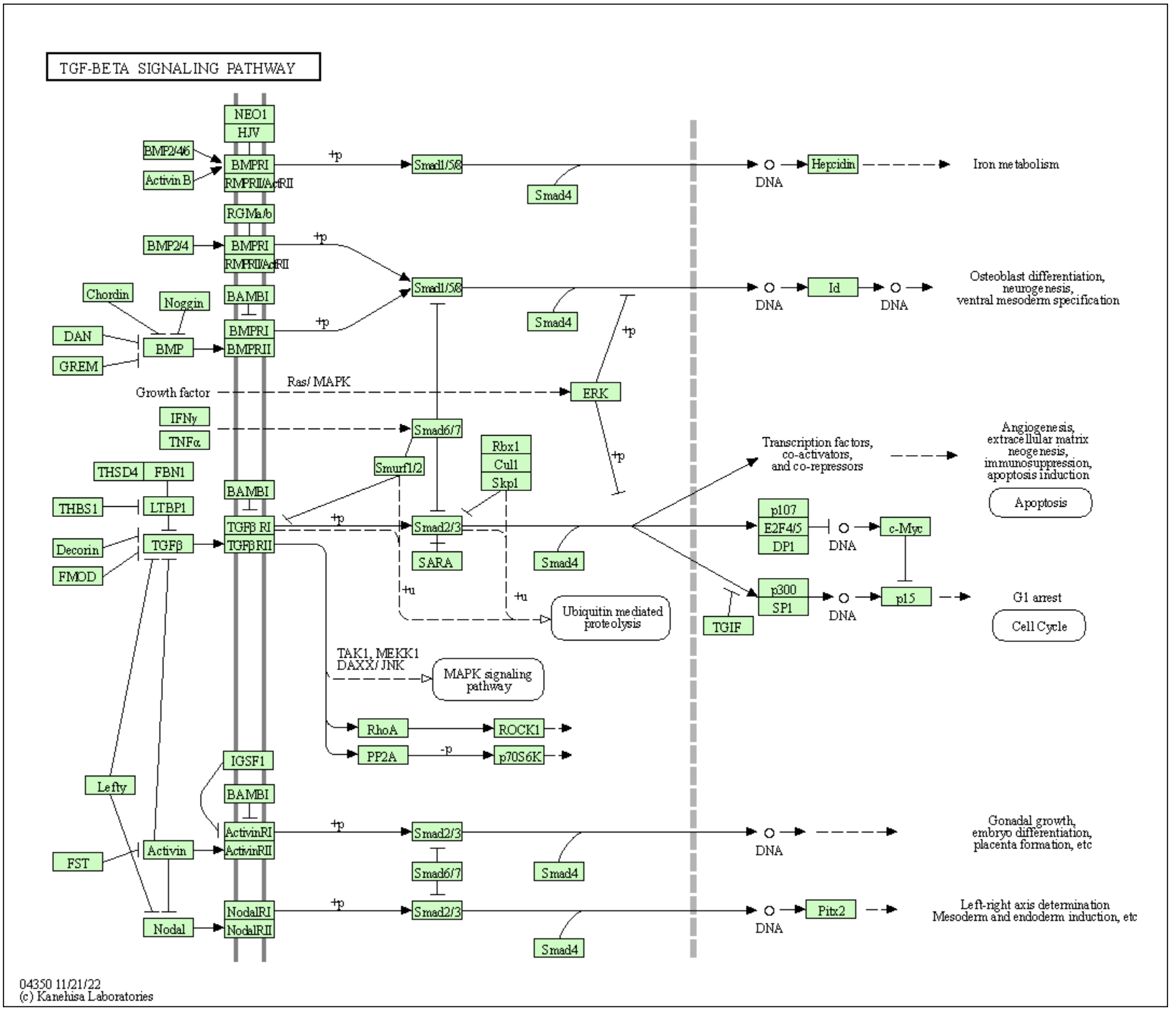


**Figure S3.** **The TGF-β pathway of SMAD family genes.**
