# Supplementary material for: The immune regulation and therapeutic potential of the SMAD gene family in breast cancer
Source: Sci Rep. 2024 Mar 21;14:6769. doi: 10.1038/s41598-024-57189-6 (PMC10958012; doi:10.1038/s41598-024-57189-6)
Supplement: Supplementary file 2 — Supplementary Table S1. [file 41598_2024_57189_MOESM2_ESM.docx]

Table S1. The expression of SMAD family genes in breast cancer.

| The SMAD family genes | p-value |
| --- | --- |
| SMAD1 | 0.001832765 |
| SMAD2 | 1.388382e-19 |
| SMAD3 | 1.726386e-23 |
| SMAD4 | 3.879854e-39 |
| SMAD5 | 2.662327e-18 |
| SMAD6 | 5.408121e-11 |
| SMAD7 | 0.01203488 |
| SMAD9 | 2.749678e-46 |
